# Comparative study between anterior symphyseal platting and percutaneous symphyseal screws for treatment of traumatic symphyseal diastasis

**DOI:** 10.1007/s00264-025-06446-y

**Published:** 2025-02-15

**Authors:** Wael Salama, Hossam Hosny, Elshazly Mousa, Moustafa Elsayed

**Affiliations:** Sohag University Hospitals (SUH), Sohag University, Sohag, Egypt

**Keywords:** Pelvic fractures, Symphysis pubis, Comparative study, Symphysis diastasis, Percutaneous screw, Symphyseal plating

## Abstract

**Purpose:**

Symphyseal diastasis accounts for 13–16% of pelvic ring injuries. Symphyseal plating via a Pfannenstiel approach was the standard method of fixation for symphysis diastasis. Recently, percutaneous reduction and fixation of pelvic fractures have been employed to treat various pelvic ring and acetabulum injuries. The current study aims to compare the clinical and radiological results of treatment of symphysis pubis diastasis using symphyseal plating and percutaneous symphyseal screws.

**Methods:**

It is a retrospective study conducted at a trauma centre at academic level I. One hundred and ten patients were identified in our records. Sixty patients were excluded according to our exclusion criteria. Fifty patients were included in this study. Among which were 26 patients treated with anterior symphyseal plating (Group A) and 24 patients treated with percutaneous symphyseal screws (Group B). Posterior pelvic injury was fixated according to the existing pathology. In both groups, we recorded operation time, intraoperative blood loss, length of the incision, number of x-ray shots, changes in symphysis distance (preoperative, immediate postoperative, and in the last follow-up), and time for union. At the last follow-up, the clinical evaluation was conducted using the Visual Analogue Scale (VAS), and the functional evaluation was conducted using the Majeed scoring method for both groups.

**Results:**

All patients have followed up for at least two years. According to the Majeed Score, group A’s functional classification was excellent for fourteen patients, good for seven, fair for two, and poor for three cases. Group B’s functional classification was excellent for seventeen patients, good for six, and poor for one. The operative time and intraoperative time were significantly different between both groups, while the symphysis diastasis at the last follow-up was insignificant. Five patients in group A showed metal failure in the form of plate breakage, screw loosening, and screw backing out. In Group B, one case showed implant failure and loss of reduction in the form of screw backing out and widening of the symphysis pubis. Two patients in group A had infections at the incision site, which were treated with antibiotics and daily dressings and resolved adequately. No recorded cases of infection in group B.

**Conclusion:**

Both techniques showed favourable results. The group with symphyseal plating showed a higher failure rate than the group with percutaneous screw fixation. The symphyseal screw group had shorter operative time, smaller incision, and less intraoperative blood loss than the symphyseal plating group but more radiation exposure. The symphyseal screw technique is a technically demanding technique and requires a high learning curve. It involves more radiation exposure, especially in inexperienced surgeons.

## Introduction

Symphyseal diastasis accounts for 13–16% of pelvic ring injuries [1]. Symphyseal diastasis, or fracture of the superior and inferior pubic rami, whether on one side or both, are signs of failure of the anterior pelvic ring [20]. Disruption of the symphysis pubis will cause pelvic ring instability and is a consistent indication of anterior pelvic ring fixation if the symphysis pubis diastasis is more than 25 mm [14, 20]. According to the Young and Burgess Classification System, symphysis diastasis results from anteroposterior compression injury (APC) [9]. Surgical treatment of unstable pelvic fractures aims to restore pelvic stability through accurate anatomic reduction and stable pelvic fixation. The stable pelvic fixation will reduce the pain and allow the patient early ambulation and early return to work. It will decrease patient morbidity, which ranges from 5 to 20% following pelvic fractures [21, 34]. Adequate reduction and fixation of the displaced symphyseal disruption is essential to restore stability and alignment of the pelvic ring. The anterior pelvic ring provides about 30% of pelvic stability [3]. Plating is the standard method of fixation of symphysis diastasis. Other methods of fixation are tension band wiring (two cancellous bone screws and wire loops) and absorbable suture materials [9, 15]. Open osteosynthesis of the symphysis pubis through conventional plates is not a complication-free technique. Its advantages are open reduction, and plating allows direct anatomic reduction, improved stability, and low morbidity and does not routinely require implant removal. Its disadvantages are extensive exposure, which may lead to complications such as blood loss, bladder injury, neural or vascular injury, wound healing problems, and heterotopic bone formation [5]. Fixation failure for symphysis plating ranges from 12 to 75%, loss of reduction occurs from 7 to 24%, and revision rates range from 3 to 9% [5, 7, 23]. To reduce these complications, several other techniques have been reported, including external pelvic fixation, percutaneous cannulated screw fixation, and minimally invasive plate osteosynthesis. Recently, percutaneous reduction and fixation of pelvic fractures have been employed to treat various pelvic ring and acetabulum injuries. The advantages of percutaneous fixation are shorter operative times, less blood loss, and lower surgical site infection rates compared to traditional open approaches [12, 21, 28]. The current study aims to compare the clinical and radiological outcomes of treatment of symphysis pubis diastasis using symphyseal plating and percutaneous symphyseal screws.

## Patients and methods

It is a retrospective study conducted at a trauma center at academic level I. All patients with symphyseal diastasis who were presented to our emergency department from September 2015 to July 2022 were included in our study. One hundred and ten patients were identified. Sixty patients were excluded according to our exclusion criteria. Fifty patients were included in this study. Scientific and ethical committees approved the study at our university. Informed written consent has been obtained from all participants. **All skeletally mature patients with symphyseal diastasis more than 25 mm were included in our study with or without posterior ring injury.**

### Exclusion criteria

1. Patients with immature skeletons.
2. Open fractures.
3. Patients who suffered from pubic rami fractures or concomitant acetabular fractures.
4. Patients with clinical and radiographic follow-up for less than two years.
5. Medical contraindications include combined neurovascular injuries and uncontrolled medical diseases.

All patients had been evaluated initially at the emergency unit, and necessary resuscitation (according to the ABCDE protocol) was done [18]. A trained orthopaedic surgeon did the initial evaluation. After that, all essential clinical and radiological assessments were done. Every patient who was diagnosed with an unstable pelvic fracture had a pelvic binder and, if necessary, a skin traction. These were short-term interventions until the patient was hemodynamically stable and ready for surgery. The patients were admitted to our pelvic and hip unit and planned for surgical intervention to fix the pelvic ring. Twenty-six patients were treated with open reduction and anterior symphyseal plating (Group A), while twenty-four patients were treated percutaneously with two partially threaded cancellous screw, 32 mm (Group B).

### Surgical technique

#### Surgical approach for symphyseal plating (Group A)

**Fig. 1:**
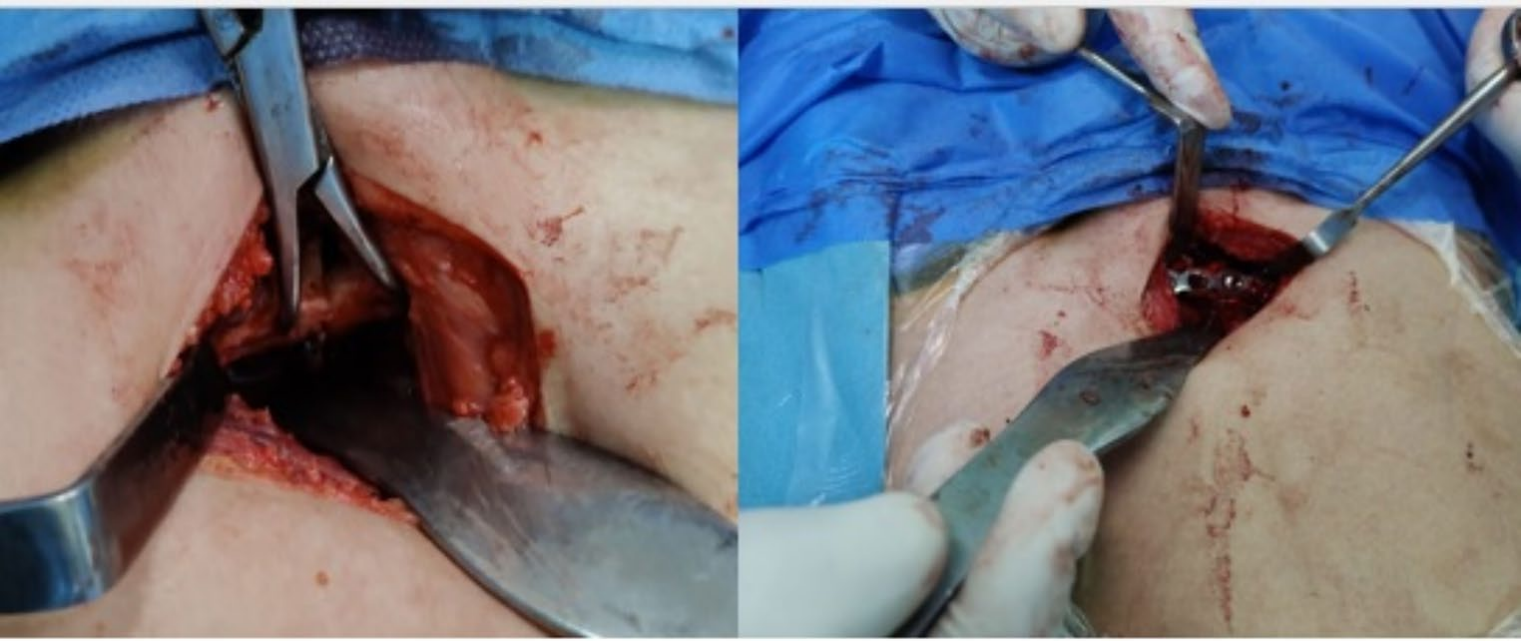
Intraoperative photo showing symphysis pubis reduction and fixation with symphyseal plate

All patients were positioned supine. All patients were treated through the Pfannenstiel approach. A big reduction clamp was used for the reduction. The clamp tips might be positioned in the medial portion of the obturator foramen or on the pubic tubercle. A four-hole or five-hole reconstruction plate was used for symphyseal fixation. Screws were inserted on either side of the symphysis using the compression mode. Figure 1 Posterior fixation was done in all cases with associated posterior pelvic ring injury to augment posterior constructs. Fixation of the posterior pelvic ring had been done either using an iliosacral screw, a lateral compression screw or bridging plates according to existing pathology posteriorly. Skin closure was done in layers. Intraoperative radiographs can be taken to confirm reduction using C-arm X-ray fluoroscopy.

### Surgical approach for symphyseal screw fixation (Group B)

**Fig. 2:**
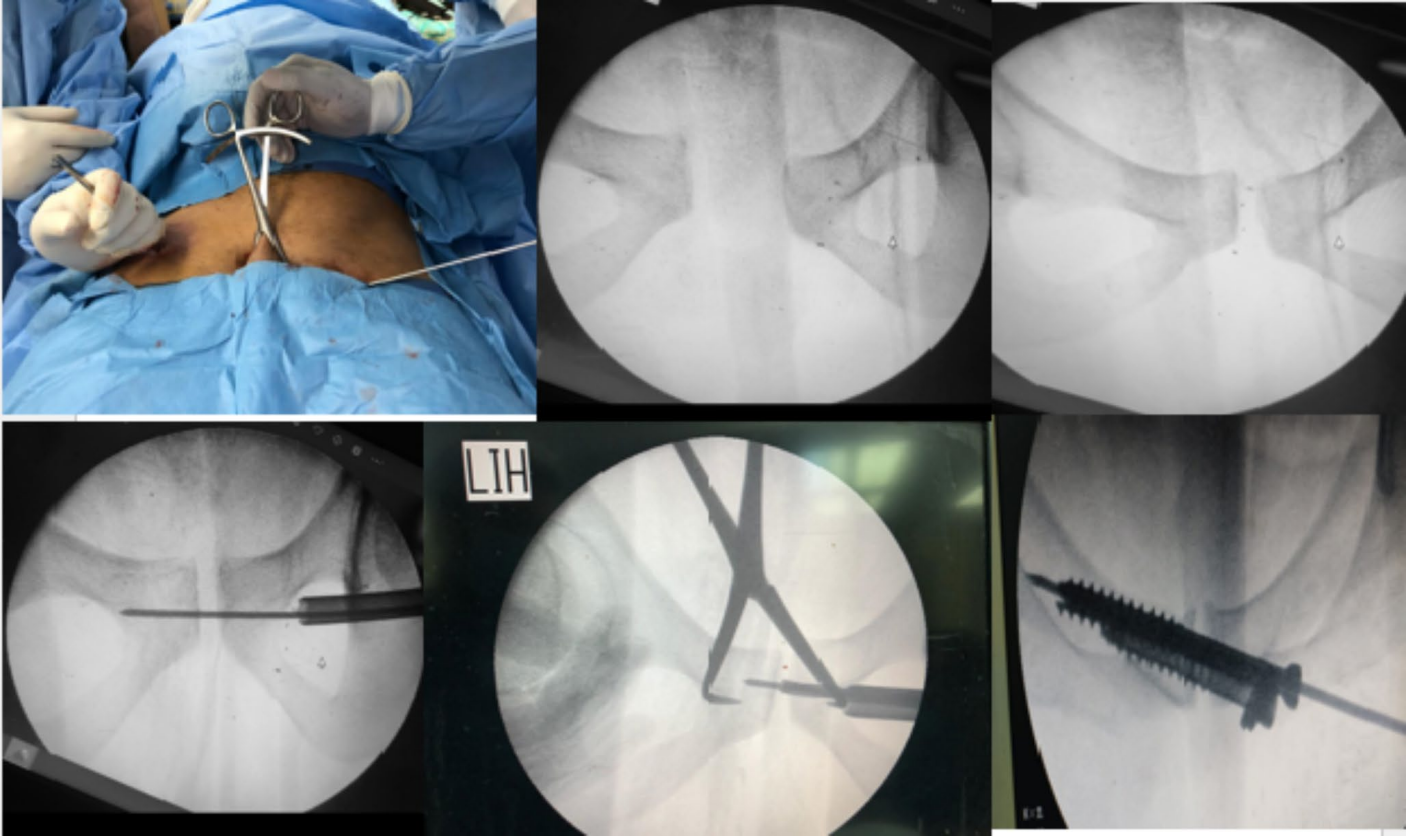
Intraoperative photo showing reduction technique for symphyseal screw fixation

**Fig. 3:**
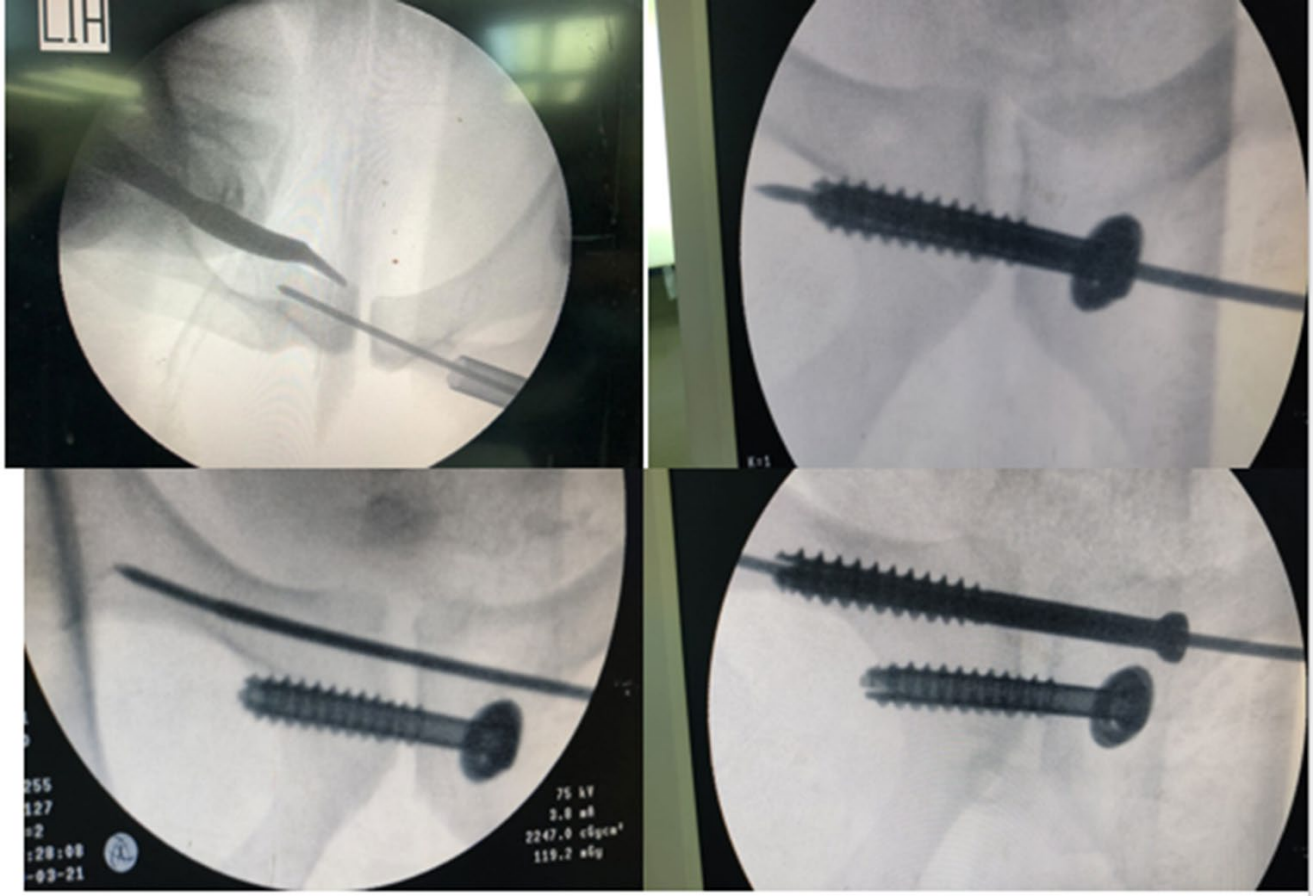
Intraoperative photo showing technique for percutaneous screw insertion

All patients were positioned supine. Two orthopaedic trauma surgeons performed all operations. C-arm X-ray fluoroscopy monitoring was used to obtain high-resolution views (anteroposterior, inlet, and outlet views). The reduction was from posterior to anterior. Fixation of the posterior pelvic ring had been done as described above. The reduction of symphysis was done by two methods: either a Schanz pin was inserted in the supra-acetabular region on the injured side and approximation of both ends of the symphysis pubis till satisfactory reduction was obtained, then applying a reduction clamp to maintain the reduction, or manually, as the assistant surgeon pushed the pelvic bones from both sides till satisfactory reduction was obtained, then applying the reduction clamp. After a satisfactory reduction was obtained, fixation had been made using two percutaneous screws. A guide wire was inserted between the superior ramus of the pubis and the pubic tubercle. It was crucial to look after the female uterus’s round ligament and the male spermatic cord. A cannulated drill bit with a protective long sleeve was introduced over the guided wire. Another guide wire was used to measure the length of the cannulated part, which equals the screw length. Fixation was accomplished by placing two partially threaded cannulated screws, 7.3 mm, crossing the symphysis in a transverse configuration and parallel to each other and from one side. Figures 2 and 3.

## Measurement and outcomes

We aimed to evaluate the clinical and radiological outcomes of treatment of symphysis pubis diastasis using both methods of fixation.

### Preoperative evaluation

Preoperative symphyseal diastasis measurement in millimeters, visual analogue score (VAS) [32], Henderson horizontal reference lines [13].

### Intraoperative evaluation

Intraoperative blood loss, operative time (from opening the incision to closing the incision), number of intraoperative x-ray shots, length of the incision, and incidence of intraoperative complications such as bladder injury or vascular injury.

### Immediate postoperative evaluation

Immediate symphyseal diastasis measurement, visual analogue score (VAS), Henderson horizontal reference lines.

### Postoperative evaluation at the last follow-up

Symphyseal diastasis, Visual Analogue score (VAS), Henderson horizontal reference lines, Majeed scoring system [16], time for union, failure of reduction, and incidence of complications as metal failure, screw or plate breakage, or screw loosening.

Evaluation of the patients was done by another orthopaedic surgeon (not an author). Measurement of symphyseal diastasis (on the PACS system) was done by a consultant radiologist. We have used Henderson horizontal reference lines to assess the pelvic vertical displacement preoperatively, immediately postoperatively and at the last follow-up visit. The preoperative plain radiographs and the immediate postoperative plain radiographs were compared to assess residual postoperative displacement, while the immediate plain radiographs and the plain radiographs at the final follow-up were compared to evaluate late displacement. Displacement more than 1 cm is considered failure. Fracture union was assessed using conventional radiological parameters, viz. bridging bone callus, obliteration of the fracture line, and cortical continuity and bridging of the fracture at three of four cortices [8].

### Postoperative protocol

Prophylactic measures against thromboembolism (low molecular weight heparin) were initiated immediately postoperatively. Follow up of cases at two weeks postoperative, six weeks, three, six and 12 months, then every year. Partial weight-bearing with crutches and physiotherapy was allowed after six weeks. Full weight-bearing started according to the follow-up.

### Statistical analysis

The data were analyzed with SPSS software (version 17.0, SPSS Inc., Chicago, IL). Data regarding symphysis pubis diastasis distance pre-operative, immediate post-operative, and at the final follow-up visit were tested using the ANOVA test. Other variables, such as operative time and blood loss, between both techniques for symphysis pubis fixation were tested by a two-sample t test. A chi-squared test was used to detect significance regarding the incidence of infection and implant failure. The Mann-Whitney U test was used to compare Majeed scores on both methods of fixation. A P value of < 0.05 was considered significant.

## Results

Fifty patients were included in this study. Forty-three patients were males (group A: 22 males—group B: 21 males). The mean age for group A patients was 35.4 years, while the mean age for group B was 37.7.

The mechanism of injury for both groups was motor vehicle accidents in 33 cases, falling from height in 11 cases, and motorcycle accidents in 6 cases. Average days for surgery were 4.11 days for group A and 4.2 days for group B. All patients have followed up for at least two years. Table 1.

**Table 1:** Demography of our study

	Group A (anterior symphyseal plating)	Group B (Percutaneous symphyseal screws)
Number of cases	26 patients	24 patients
Age	Range from 22 to 61 mean age = 35.4	Range from 20 to 58 mean age = 37.7
Sex	Males 22 cases (84.6%) Female 4 cases (15.4%)	Males 21 cases (87.5%) Female 3 cases (12.5%)
Mode of trauma	Motor car accidents in 18 cases (69.2%) Falling from height in six cases (23%) Motorcycle accidents in two cases (7.8%)	Motor car accidents in 15 cases (62.5%) Falling from height in five cases (20.8%) Motorcycle accidents in four cases (16.7%)
AO-OTA classification	AO/OTA 61-B 5 cases (19.3%) AO/OTA 61-C 21 cases (80.7%)	AO/OTA 61-B 3 cases (12.5%) AO/OTA 61-C 21 cases (87.5%)
Type of posterior ring injury	Trans-sacral injury in 10 cases (38.5%) Trans iliac fracture in 6 cases (23.1%) Sacroiliac joint dislocation in 5 cases (19.2%) open book injury with intact posterior pelvic ring in 5 cases (19.2%)	Trans-sacral injury in 9 cases (37.5%) Trans iliac fracture in 7 cases (29.2%) Sacroiliac joint dislocation in 5 cases (20.8%) open book injury with intact posterior pelvic ring in 3 cases (12.5%)
Time to surgery	Range from 2 to 6 days. Average 4.11 days	Range from 2 to 7 days. Average 4.2 days

In group A, a four-holed 4.5 mm reconstruction plate was used for fixation in twenty patients while a five-holed 4.5 mm reconstruction plate was used in six cases. Regarding posterior pelvic injury, iliosacral screw was used in 15 cases, anterior iliac plating in three cases, and lateral compression screw was used in three cases (two cases from the anterior approach and one case from the posterior approach).

In group B, two partially threaded cancellous screws (diameter 7.3 mm) were used for fixation of the anterior pelvic ring in all cases. Regarding posterior pelvic injury, iliosacral screw was used in 14 cases, anterior iliac plating in two cases, and lateral compression screw was used in five cases (3 cases from the anterior approach and 2 cases from the posterior approach).

Our results were statistically significant regarding operative time, intraoperative blood loss, number of intraoperative x-ray shots, length of incision, and vertical pelvic displacement according to Henderson horizontal reference lines. Table 2.

**Table 2:** Intraoperative and postoperative outcomes

	Group A (anterior symphyseal plating)	Group B (Percutaneous symphyseal screws)	*P-Value*
Operation time (Min)	58.3	42.3	0.0001
Intraoperative blood loss (ml)	182.69	10.95	0.00001
Number of intraoperative x ray shots	15 ± 2	95 ± 15	0.00001
Length of incision (cm)	9 ± 3	2 ± 1	0.00001
Majeed scoring system	Excellent 14 cases Good 7 cases Fair 2 cases Poor 3 case	Excellent 17 cases Good 6 cases Fair 0 cases Poor 1 case	---------
Henderson lines	4 cases of vertical migration	No case of vertical migration	0.043
Time of union(days)	45.4	44.1	0.10768

The mean pre-operative VAS score was 9 ± 1 for group A, while the immediate mean post-operative score was 4.5 ± 1. The mean postoperative score at a minimum of two years of follow-up was 1.4 ± 0.5.

The mean pre-operative VAS score was 9 ± 1 for group B, while the immediate mean post-operative score was 4 ± 1. The mean postoperative score at a minimum two-year follow-up was 1.1 ± 0.2.

**Fig. 4:**
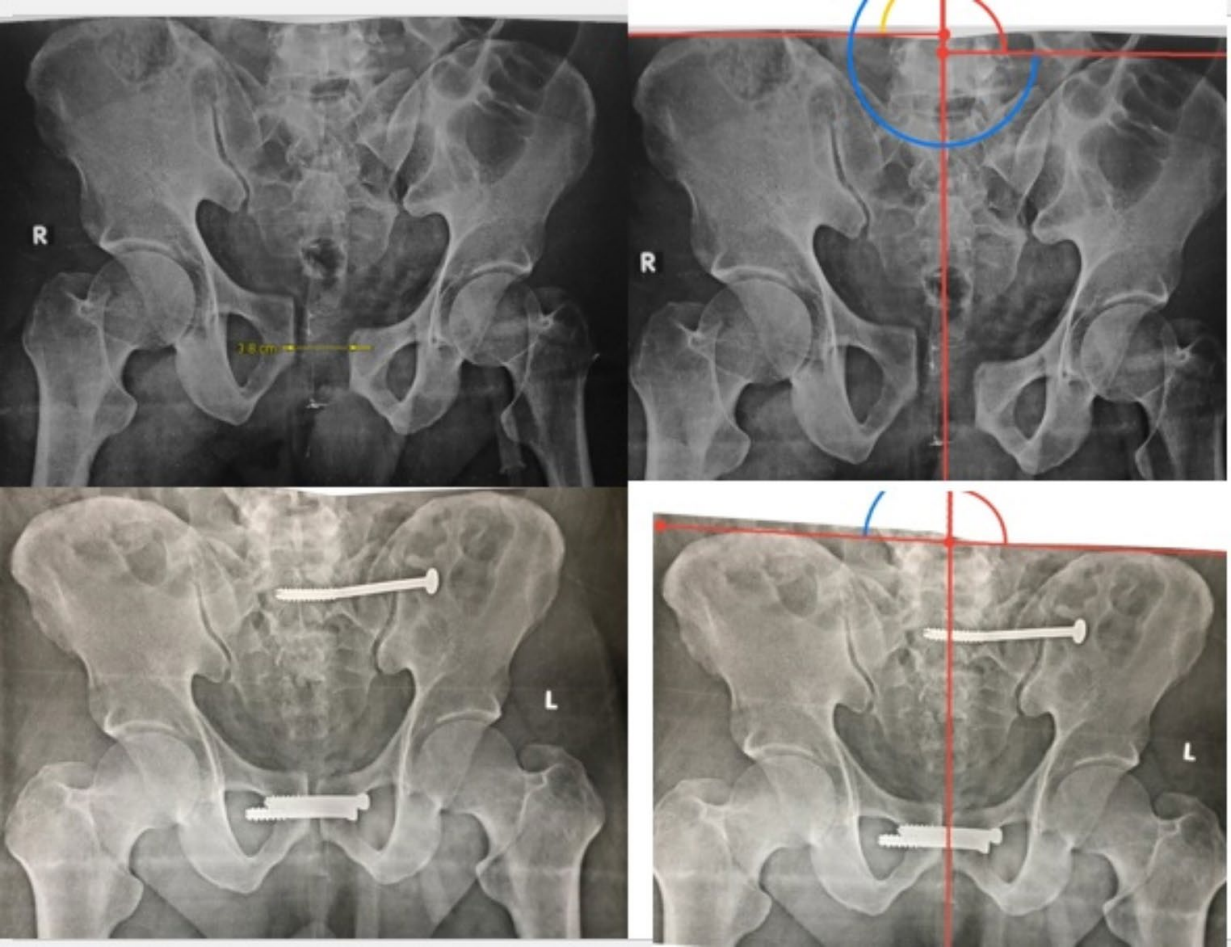
Preoperative and postoperative plain radiographs showing symphyseal screw fixation with measurement of vertical pelvic displacement using Henderson horizontal reference lines

**Fig. 5:**
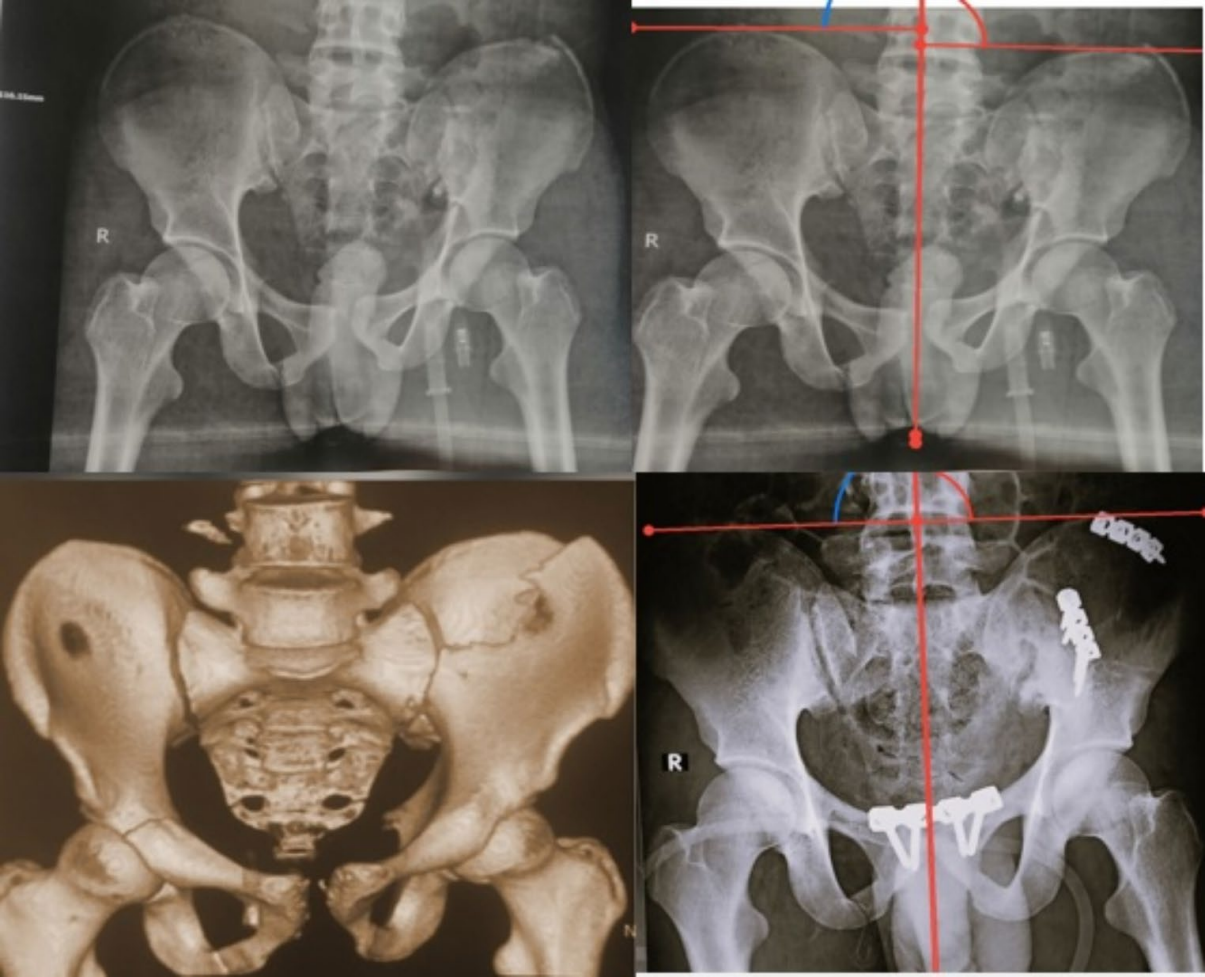
Preoperative and postoperative plain radiograph showing symphyseal plate fixation with measurement of vertical pelvic displacement using Henderson horizontal reference lines

The mean post-operative symphyseal diastasis at the last follow-up for group A was 0.869, while the mean post-operative symphyseal diastasis at the last follow-up for group B was 0.612. (P value was insignificant = 0.15117). Figures 4 and 5.

There is no statistically significant difference between both groups regarding the Majeed scoring system.

Using Henderson horizontal reference lines, four cases in group A had vertical displacement of more than 1 cm at the last follow-up visit, while there were no recorded cases of vertical displacement in group B. Table 3.

**Table 3:** Functional and radiological outcomes

	Symphysis diastasis in cm		Visual analogue Score (VAS)		Henderson horizontal reference lines in cm	
	Group A Symphyseal plating	Group B Symphyseal Screws	Group A Symphyseal plating	Group B Symphyseal Screws	Group A Symphyseal plating	Group B Symphyseal Screws
Preoperative	3.5	3.26	9.2	9.5	1.9	1.8
Immediate Postoperative	0.415	0.45	4	4.5	0.36	0.35
Postoperative at last follow up	0.869	0.612	1.4	1.1	0.65	0.3
P value Postoperative at last follow up between both groups	0.15117		0.0152		0.01879	

P value for immediate and Postoperative at last follow up Henderson horizontal reference line for symphyseal plate (Group A) was 0.04409

P value for immediate and Postoperative at last follow up Henderson horizontal reference line for symphyseal screws (Group B) was 0.86901

**Fig. 6:**
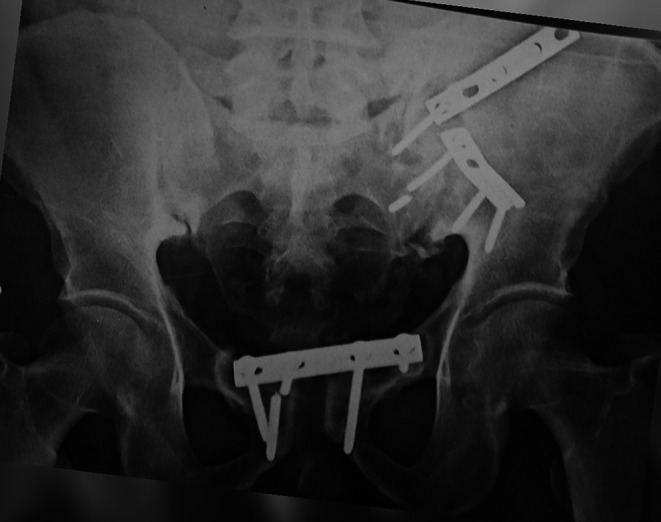
Last follow up plain radiograph showing metal failure of symphyseal plate

Five patients in group A (19%) showed metal failure in the form of plate breakage, screw loosening, and screw backing out. Plate removal was done in three patients (11.5%), and the other two patients refused another surgery. Figure 6.

**Fig. 7:**
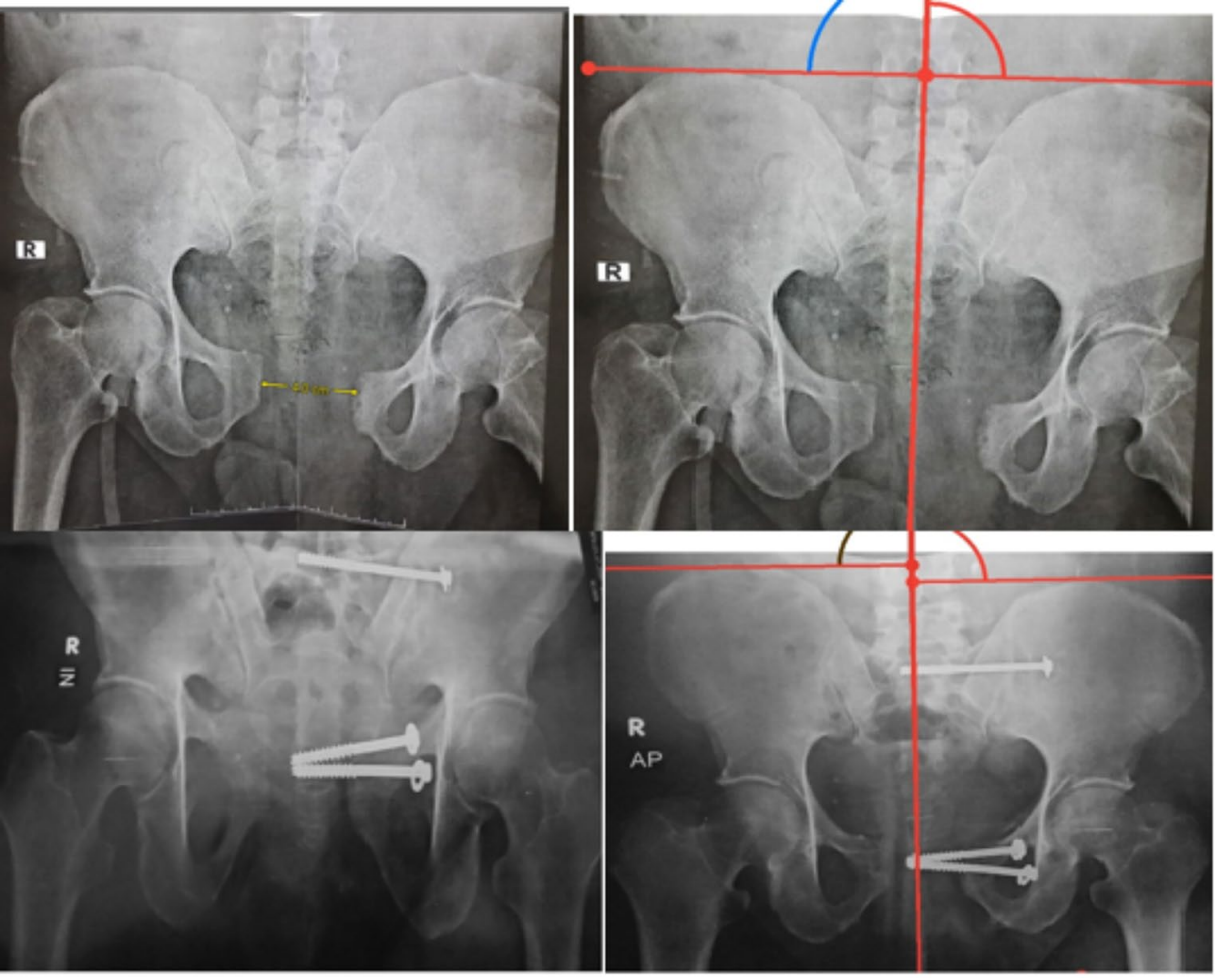
Preoperative and last follow up plain radiographs showing a failed case of symphyseal screw fixation with measurement of vertical pelvic displacement using Henderson horizontal reference lines

In Group B, one case showed implant failure (4%) and loss of reduction in the form of screw backing out and widening of the symphysis pubis at three months postoperatively. Screw removal was done, and application of external fixators for one month, and the patient refused to do another surgery. Figure 7.

Two patients in group A (7.5%) had infections at the incision site, which were treated with antibiotics and daily dressings and resolved adequately. No recorded cases of infection in group B. Table 4.

**Table 4:** Comparison of complications

	Group A (anterior symphyseal plating)	Group B (Percutaneous symphyseal screws)	*P -Value*
Metal failure (Screw loosing or backing out)	5 cases (19%)	1 case (4%)	0.005
Infection	2 cases (7.5%)	0	0.326
Removal of fixation construct	3 (11.5%)	1 case (4%)	0.1613

## Discussion

Open reduction and internal fixation have become the standard and preferred method for fixing pelvic and acetabular fractures. Symphyseal plating via a Pfannenstiel approach is the standard method of fixation of symphysis diastasis. Advantages of open reduction and fixation are direct anatomic reduction, low morbidity, low infection rate, low incisional hernia rates, and does not routinely require implant removal [6, 12, 17]. Its disadvantages are extensive exposure, which may lead to complications such as blood loss, neurovascular injury, wound healing problems, heterotopic bone formation, lateral incision extension in large obese patients, and this can increase the risk of damage to inguinal canal and its contents. One of the advantages of open pelvic approaches is the removal of retropubic haematoma caused by traction of the prevesical venous plexus; however, in percutaneous pelvic techniques, the retropubic hematoma was probably always absorbed without consequences, and it did not affect our results [6, 12, 17].

Percutaneous procedures, which aim to achieve anatomical reduction and shrink incisions, have recently been developed and are expected to lower open procedures’ dangers [29]. Percutaneous screw fixation in pelvic fractures has replaced traditional methods of pelvic ring fixation. The Percutaneous technique for insertion of the iliosacral screw was first described by Rout et al. [22]. The advantages of percutaneous fixation of symphysis diastasis include less blood loss, minimal invasiveness with small incision size, and short operative time with experienced surgeons. Disadvantages are excess exposure to radiation with using an intraoperative C-arm, long operative time in learning surgeons, and the need for experienced surgeons.

Traditional open reduction and internal fixation using reconstruction plates have been widely used for traumatic symphysis pubis diastasis [24, 25]. Biomechanical studies have reported that symphyseal plating is an effective method to stabilize the anterior pelvic ring [27], and satisfactory outcomes have been confirmed in published case series [10, 11, 26]. There are recent clinical trials describing the fixation of symphyseal diastasis using percutaneous screws and recommending percutaneous screws for symphysis pubis fixation [5, 9, 17, 34].

The symphysis pubis is a fibrocartilaginous joint. It consists of a disc that is positioned between the articular surfaces of the pubic bones. Under physiological settings, it can move a little bit, often by up to 2 mm and rotating 1 degree in adults. In multiparous females in particular, a larger degree of mobility can be observed [2]. Some authors stated that normal physiological movement is restricted when the pubic symphysis is rigidly fixed. The metalwork of the symphysis may eventually be stressed by physiological micro-motion, which could lead to implant failure like screw loosening or plate breaking and symphyseal diastasis. Therefore, it appears that a significant implant failure rate with a low revision rate is a unique characteristic of the plating of traumatic symphysis diastasis [2, 30].

O’Neill et al. [19] in their biomechanical study to compare percutaneous screw fixation versus plate osteosynthesis for fixation of pubic symphysis diastasis, they reported that symphyseal screws may be a viable alternative to classically described symphyseal plating.

Zheng et al. [35] evaluated the biomechanical characteristics of seven fixation methods to treat symphysis pubis diastasis using finite element analysis. They concluded that dual cannulated screw fixation is the best fixation method for traumatic symphysis pubis diastasis that offers ideal outcomes to maintain stability and prevent failure biomechanically. The single plate with crossed symphyseal screws and dual symphyseal plates methods had a better and more effective fixation method than single plate fixation.

The intramedullary method of cannulated screw fixation is responsible for its biomechanical advantage. It was previously established that intramedullary nailing had a lower failure rate than a plate and could lessen the implant’s stress load. To prevent implant failure, care had to be taken precisely where the stress was concentrated at the screw-plate contact site and in the middle of the cannulated screw [31].

In their biomechanical study, Yao et al. compared fixation of symphysis diastasis using a single plate (4-hole and 5-hole), double plates (4-hole and 5-hole), and a single screw (cannulated screw 7.3 mm diameter, double parallel screw (cannulated screw 6.5 mm diameter), and double-crossed screw (cannulated screw 6.5 mm diameter). They reported that single or double plate fixation achieved greater stiffness than single screw fixation, while double screw fixation, either in parallel configuration or crossed configuration, achieved greater stiffness than single or double plating techniques. They concluded that stabilization of the posterior pelvic ring is mandatory to restore overall pelvis stiffness [33].

Cano-Luis et al. performed a cadaveric biomechanical study to assess the use of two cannulated screws with a 6.5 mm diameter for the fixation of Tile B1 and B3 fractures. They compared the displacement of the symphysis pubis of the normal and injured pelvis (fixed with screws) under 300 N and found no difference, and the injured pelvis fixed with two screws can resist displacement and rotational forces [4].

Yu et al. performed a biomechanical study and compared fixation of symphysis diastasis with a single screw and traditional anterior plating. They found a lower maximal displacement with plating compared to single screw fixation. Their retrospective case-control study compared symphysis diastasis fixation in Tile B1 patients treated with single percutaneous screw fixation (24 patients) and traditional symphysis pubis plating (27 patients). They concluded that the two groups had no significant difference in implant failure, wound infection, or revision surgery. They observed a significant improvement in intraoperative blood loss, operative time, and scar length in the percutaneous screw fixation group [34]. Our results were similar to Yu et al. regarding significant improvement in intraoperative blood loss and operative time, but in our study, we had more implant failure in the symphyseal plate group than in the group of symphyseal screw group.

By comparing the clinical and radiological results of both groups, we found that both methods of fixation had the same functional outcome with relative superiority of percutaneous symphyseal screw group (group B) over symphyseal plating group (Group A) regarding the Majeed scoring system. This is attributed to the increased failure rate in the symphyseal plate group (five cases, 19%). The visual analogue score (VAS) was statistically significant for both groups. This is explained as fracture stability; either by plate or screw, will actually reduce the pain and improve clinical symptoms.

Regarding radiological evaluation or fracture union (in cases with posterior pelvic injury), there was no statistical difference between the two groups. Both groups had a close ratio (the symphyseal plate group was 45.4 days while the symphyseal screw group was 44.1 days). We depend on Henderson horizontal reference lines to evaluate vertical displacement of pelvic fractures. Comparing the preoperative and immediate postoperative measurements for each group was statistically significant, and this represents the residual displacement or adequacy of reduction. While comparing the immediate and last follow-up measurements, we can see the incidence of the failure rate. It was insignificant for group B, but it was significant for group A, and this was affected by the incidence of failure rate in group A.

Comparing the postoperative measurement at last follow-up for both groups was statistically significant, and this means that the failure rate of symphyseal plating (Group A) is higher than that of symphyseal screws (Group B).

The most observed differences between both groups are the amount of intraoperative blood loss, operative time, length of incision, number of x-ray shots (representing x-ray radiation exposure), and vertical displacement of pelvic fracture. It was highly significant between both groups. The small amount of intraoperative blood loss in symphyseal screw fixation (Group B) is attributed to the small incision of screw insertion compared to the large incision needed in the symphyseal plate group (Group A) to do the reduction of symphysis diastasis and place the four holed or five holed reconstruction plate. The operative time was long in the first three cases in the symphyseal screw group (Group B), and it shortens gradually with increased surgeon experience and familiarity with the technique, and finally the operative time in the symphyseal screw group (42.3 min) is shorter and significantly less than in the symphyseal plate group (Group A) (58.3 min).

The surgery with symphysis pubis reduction and percutaneous fixation of screws is dependent mainly on intraoperative C-arm X-ray fluoroscopy, hence more radiation exposure than symphysis pubis plating. However, this is one of the limiting factors for the use of percutaneous screw fixation. Small-sized skin incision is one of the percutaneous technique benefits (Group B). It was statistically significant between both groups.

Wound infection was observed in two cases in the symphyseal plate group, while no cases of wound infection were observed in the symphyseal screw group. This can be explained by two causes: the incision size and long operative time in the symphyseal plate group. These may contribute to the relatively high rate of infection in the symphyseal plate group. Our results were similar to Mu et al. [17], Chen et al. [5], and Yu et al. [34]. They reported less intraoperative blood loss and shorter operative time and skin scar in cases of percutaneous symphyseal screw fixation.

Both methods are valid for fixation of traumatic symphysis diastasis. Both techniques yielded similar functional outcomes. Metal failure was more evident in patients treated with symphyseal plating (5 cases (19%)), and this was accompanied by more increases in vertical pelvic displacement. The percutaneous screw technique (group B) has the advantage of a short operative time, less blood loss, and minimally invasive surgery with smaller incision size than the symphyseal plating techniques. Despite the technique having the advantage of being minimally invasive, the high exposure to radiation and long operative time are considered limiting factors for applying the new technique. However, with increasing the learning curve and the experience of the operating surgeon, the time for operation and the amount of radiation exposure decreased gradually. These are the main reasons making the orthopaedic surgeons not perform percutaneous reduction and screw fixation of symphysis pubis diastasis. Patients with abdominal herniation, open wounds, irritable testis, bladder injury, and surgical site skin infection are contraindications for percutaneous symphyseal screw fixation [17]. **Gentle closed reduction and a persistent urine catheter can help to avoid bladder incarceration during percutnaous symphseal pubis reduction.**

Limitations of the study include a small sample size. Despite we had a satisfactory result compared to the existing studies in the literature, we encourage other authors to perform large prospective randomized trials comparing all types of symphyseal diastasis fixation to have more data on the best method of stabilization of symphyseal disruption.

## Conclusion

Both techniques showed favorable results. The group with symphyseal plating showed a higher failure rate than the group with percutaneous screw fixation. The symphyseal screw group had shorter operative time, smaller incision, and less intraoperative blood loss than the symphyseal plating group but more radiation exposure. The symphyseal screw technique is a technically demanding technique and requires a high learning curve. It involves more radiation exposure, especially in inexperienced surgeons.

## Data Availability

All data and materials are available from the corresponding author on reasonable request.

## Abbreviations

VAS: Visual analogue scale
APC: Anteroposterior compression injury
ABCDE protocol: Airway. Breathing, Circulation, Disability, and Exposure
PACS: Picture archiving and communication system
